# Complete genome sequences of three effective nitrogen-fixing strains of *Bradyrhizobium ottawaense* from Canada

**DOI:** 10.1128/mra.00535-26

**Published:** 2026-09-14

**Authors:** Eden S. P. Bromfield, Sylvie Cloutier

**Affiliations:** 1 Agriculture and Agri-Food Canada, Ottawa, Ontario, Canada; Rochester Institute of Technology, Rochester, New York, USA

**Keywords:** *Bradyrhizobium ottawaense*, complete genome, nitrogen fixation, plant growth-promoting genes, glyphosate, soybean, Canada

## Abstract

We report complete genome sequences of three nitrogen-fixing *Bradyrhizobium ottawaense* strains isolated from soybeans in Canada. Each ~9.0 Mb genome (chromosome and plasmid) harbors predicted genes for nodulation, nitrogen fixation, N_2_O mitigation, phosphate solubilization, iron acquisition, phytohormone production, and stress tolerance, highlighting their potential for sustainable agriculture.

## ANNOUNCEMENT

The genus *Bradyrhizobium* includes economically important bacteria that fix atmospheric nitrogen in symbiosis with soybeans. The strains were isolated in 1999 from surface-sterilized soybean root nodules collected in Ottawa, Canada (45°23′09.0″N, 75°43′11.0″W) (1). Pure cultures from single colonies grown on yeast extract-mannitol (YEM) agar (1) were maintained in 20% glycerol at −80°C. Phylogenetic analysis of housekeeping gene sequences placed several strains in a distinct taxon, subsequently described as *Bradyrhizobium ottawaense* (2, 3). The species type strain, OO99^ᵀ^, is effective for nitrogen fixation with soybeans (1) and efficiently reduces the greenhouse gas, nitrous oxide, to dinitrogen gas (4). In plant tests using soybeans (var. Dale) grown in Leonard jars containing nitrogen-free nutrient solution (5, 6), strains OM27, OO86, and OO100 were confirmed as effective nitrogen fixers (Table 1).

**TABLE 1:** Characteristics of genome sequences of *Bradyrhizobium ottawaense* strains OM27, OO86, and OO100 versus type strain, *B. ottawaense* OO99^T^

Characteristic		OM27	OO86	OO100	OO99^T^
Sequencing metrics					
Total raw (polymerase) reads; assembly coverage		232,719; 234×	237,870; 235×	264,796; 257×	na
Filtered subreads (number; mean, bp; N50, bp)		232,719; 9,023; 10,604	237,870; 8,964; 10,933	264,796; 8,716; 10,447	na
Genome characteristics^*a*^					
Genome size (bp) (no. contigs)		8,956,997 (2)	8,978,882 (2)	8,973,456 (2)	8,589,152 (1)
Replicon accession no. (size, bp)					
Chromosome		CP150123 (8,595,668)	CP150476 (8,608,740)	CP150125 (8,611,648)	CP029425 (8,589,152)
Plasmid^*b*^		CP150124 (361,329)	CP150477 (370,142)	CP150126 (361,808)	na
Genes (total)		8,460	8,478	8,474	8,017
CDSs (total)		8,064	8,084	8,063	7,741
G + C content % (total; chromosome; plasmid)		63.5; 63.8; 60.6	63.5; 63.8; 60.7	63.5; 63.8; 60.6	63.8; 63.8; na
Annotation features					
Predicted rRNAs (5S, 23S, 16S)		1, 1, 1	1, 1, 1	1, 1, 1	1, 1, 1
Predicted tRNAs		50	51	51	51
Predicted nodulation genes^*c*^		*nodD1D2ABCISU*(x2*)Z*; *nopP*	*nodD1D2ABCISU(x2)Z*; *nopP*	*nodD1D2ABCISU(x2)Z; nopP*	*nodD1D2ABCISU(x2)Z; nopP*
Predicted nitrogen fixation genes^*c*^		*nifHDKENBQSXAWZT*; *fixA*(x2)*B*; *ccoNOPG*(x2)*S*; *fixH*(x2*)LJ*; *fdxAB*; *nifU*; *rpoN*(x2); *modABC*	*nifHDKENBQSXAWZT; fixA*(x2)*B; ccoNOPG*(x2)*S*; *fixH*(x2)*LJ*; *fdxAB*; *nifU*; *rpoN*(x2); *modABC*	*nifHDKENBQSXAWZT; fixA*(x2)*B*; *ccoNOPG*(x2*)S*; *fixH*(x2)*LJ*; *fdxAB*; *nifU*; *rpoN*(x2); *modABC*	*nifHDKENBQSXAWZT*; *fixA*(x2)*B*; *ccoNOPG*(x2*)S*; *fixH*(x2)*LJ*; *fdxAB; nifU*; *rpoN*(x2); *modABC*
Predicted type III secretion system (T3SS) genes		*rhcJLRTVQN*	*rhcJLRTVQN*	*rhcJLRTVQN*	*rhcJLRTVQN*
Predicted conjugative type IV secretion system (T4SS) genes		*traG*; *trbI/virB10*(x3); *trbB* In plasmid: *virB1*; *trbC/virB2*; *VirB3-11*; *trbI/virB10*	*traG*; *trbI/virB10*(x3); *trbB* In plasmid: *virB1; trbC/virB2*; *VirB3-11*; *trbI/virB10*	*traG; trbI/virB10*(x3); *trbB* In plasmid: *virB1*; *trbC/virB2*; *VirB3-11*; *trbI/virB10*	*traG*(x2); *trbI/virB10*(x3); *trbB*(x3); *trbC*/*virB2*(x2); *virB3*(x2); *trbE*(x2); *trbJ*(x2); *trbK-alt*(x2); *trbLFG*
Predicted hydrogen uptake genes		*hupFNS*; *hypABCDE*; *nikR*; *rpoN*(x2)	*hupFNS*; *hypABCDE*; *nikR*; *rpoN*(x2)	*hupFNS*; *hypABCDE*; *nikR*; *rpoN*(x2)	*hupFNLS*; *hypABC*(x2)*DE*; *nikR; rpoN*(x2)
Predicted denitrification genes		*nosZDL*; *napED*(x2)*A*(x2)*C*(x2); *nirK*; *norCBQ*	*nosZDL*; *napED*(x2)*A*(x2)*C*(x2); *nirK*; *norCBQ*	*nosZDL*; *napED*(x2)*A*(x2)*C*(x2); *nirK*; *norCBQ*	*nosZDL*; *napED*(x2)*A*(x2)*C*(x2); *nirK*; *norCBQ*
Predicted phosphate solubilizing genes (locus tags)		*phoD*(x3); *phoRB*; *pqqA*(x2)*BCDE*; *gdh*; *pstS*(x2)*CAB*; *ppk2* (x2); *ppx*; inorganic phosphate transporter (*pit*?) (x2: WKW52_27140 and WKW52_23045); alkaline phosphatase (*phoX*?) (WKW52_00630)	*phoD*(x3); *phoRB*; *pqqA*(x2)*BCDE*; *gdh*; *pstS*(x2)*CAB*; *ppk2* (x2); *ppx*; inorganic phosphate transporter (*pit*?) (x2: WKW65_27220 and WKW65_23120); alkaline phosphatase (*phoX*?) (WKW65_00630)	*phoD*(x3); *phoRB*; *pqqA*(x2)*BCDE*; *gdh*; *pstS*(x2)*CAB*; *ppk2* (x2); *ppx*; inorganic phosphate transporter (*pit*?) (x2: WKW75_13395 and WKW75_17485); alkaline phosphatase (*phoX*?) (WKW75_39955)	*phoD*(x3); *phoRB*; *pqqA*(x2)*BCDE; gdh*; *pstSCAB*; *ppk2*; *ppx*; inorganic phosphate transporter (*pit*?) (x2: CIT37_38480 and CIT37_33855); alkaline phosphatase (*phoX*?) (CIT37_11835)
Predicted iron acquisition genes		*tonB*; *exbB*(x2); *exbD*(x2); *fsrB*; *Bfr*	*tonB*; *exbB*(x2); *exbD*(x2); *fsrB*; *Bfr*	*tonB*; *exbB*(x2); *exbD*(x2); *fsrB*; *Bfr*	*tonB*; *exbB*(x2); *exbD*(x2); *fsrB*; *Bfr*
Predicted phytohormone genes		*trpABCDE*	*trpABCDE*	*trpABCDE*	*trpABCDE*
Predicted stress-related genes		*acdS*; *Lrp/AsnC*	*acdS*; *Lrp/AsnC*	*acdS*; *Lrp/AsnC*	*acdS; Lrp/AsnC*
Predicted insertion sequences (total)		158 In plasmid: 90	157 In plasmid: 81	161 In plasmid: 90	170
Symbiotic characteristics					
Relative effectiveness (RE %)^*d*^		112	93	111	119

^*a*^ Predicted genes or insertion sequences are chromosomally located unless otherwise specified.

^*b*^ PATRIC/BV-BRC subsystem analysis (7) indicates that plasmid sequences of novel strains harbor genes assigned to the categories of membrane transport, metabolism, cell envelope, and DNA processing. Pairwise FastANI analysis indicates that these plasmids are nearly identical, exhibiting ≥99.97% average nucleotide identity.

^*c*^ All predicted nodulation and nitrogen fixation genes in novel strains are organized identically to reference strain OO99^T^.

^*d*^ RE (%) = ([*x* − *x*_0_]/[*x*_*e*_ − *x*_0_]) × 100, where *x*, *x*_0_, and *x*_*e*_ represent mean shoot dry weights of soybean plants (var. Dale) inoculated with test strains, uninoculated controls, and *B. diazoefficiens* USDA 110 (effective control), respectively (*n* = 4 replicates; 2 plants/replicate). na, not applicable.

For genomic analysis, bacterial strains were grown on YEM agar (7 days, 28°C), harvested, and washed. Genomic DNA was extracted (Wizard SV, Promega), purified (DNeasy PowerClean Pro, Qiagen) following standard protocols, and quantity/quality assessed using Qubit (Thermo Fisher) and DropSense 16 (Trinean, Belgium). Libraries were prepared with the SMRTbell Prep Kit 3.0 following the manufacturer’s protocol after Megaruptor 3 shearing (Diagenode) without size selection. Sequencing was performed at Genome Québec (Canada) on a PacBio Sequel II platform (30 h movie). HiFi reads were generated using default Circular Consensus Sequencing with automatic adapter trimming and read quality control. *De novo* assembly was performed using the Microbial Assembly application (SMRT Link v11.0), which circularized all replicons.

Genomes were annotated using the NCBI Prokaryotic Genome Annotation Pipeline v6.7. Sequencing metrics are summarized in Table 1. All bioinformatic tools utilized default parameters.

FastANI v1.34 (8) values for novel strains versus *B. ottawaense* OO99^T^ (2, 3) were ≥99.55%, demonstrating their high genomic similarity and assignment to the species *B. ottawaense*.

Table 1 presents genomic features of novel strains compared to *B. ottawaense* OO99^T^. The genome of each novel strain comprises a chromosome (~8.6 Mb) and a plasmid (~0.4 Mb); plasmids were identified by the presence of predicted *repABC* genes (9). All strains harbor predicted nodulation and nitrogen fixation genes, as well as type III (T3SS) (3, 10) and conjugative type IV (T4SS) (11) secretion system genes, which may influence host specificity and symbiotic performance. Predicted genes for iron acquisition (*tonB*, *exbBD*), phosphate solubilization (*pho*, *pqq*, *pst*), stress tolerance (*acdS*), and phytohormone production (*trp*) (12, 13) were identified. The detection of a predicted *nos* denitrification cluster in novel strains suggests potential for greenhouse gas (N_2_O) mitigation (4). While hydrogen uptake genes were predicted in all genomes, the novel strains lack the structural *hupL* gene found in OO99^T^, suggesting an inability to recycle hydrogen from nitrogen fixation (14). Analysis of predicted amino acid sequences using the EPSPSClass web server (15) identified class II 5-enolpyruvylshikimate-3-phosphate synthase enzymes in all strains, suggesting glyphosate resistance. Furthermore, multiple insertion sequences identified by ISEScan (16) suggest genome plasticity and potential for environmental adaptation (17).

These high-quality assemblies provide a valuable resource for genomic studies of *B. ottawaense* strains with significant potential for sustainable agriculture.

## Data Availability

The whole-genome shotgun projects for *B. ottawaense* strains OM27, OO86, and OO100 were deposited at DDBJ/ENA/GenBank as assembly accession numbers GCA_038449195.1, GCA_037997155.1, and GCA_038449185.1, respectively. Raw PacBio data were deposited in the NCBI Sequence Read Archive as SRX25699943, SRX25699941, and SRX25699930 in BioProject accession numbers PRJNA1091655, PRJNA1091664, and PRJNA1091665, respectively. *B. ottawaense* strains OM27, OO84, and OO100 were deposited in the BCCM/LMG Bacteria Collection, University of Ghent, Belgium, under the culture collection numbers LMG 34603, LMG 34605, and LMG 34607, respectively.
